# Intercostal hemangioma: Case report of a rare chest wall tumor in childhood

**DOI:** 10.1016/j.ijscr.2019.06.026

**Published:** 2019-06-20

**Authors:** Hatem Elbawab, Farouk Alreshaid, Tariq Hashem, Asayil Alnasser, Raja Husain, Yasser Aljehani

**Affiliations:** a Division of General Thoracic Surgery, Department of Surgery, King Fahad Hospital of the University, College of Medicine, Imam Abdulrahman Bin Faisal University, Dammam, Saudi Arabia; b Department of Pathology, King Fahad Hospital of the University, College of Medicine, Imam Abdulrahman Bin Faisal University, Dammam, Saudi Arabia

**Keywords:** Chest wall tumor, Chest wall reconstruction, Intercostal hemangioma

## Abstract

**Introduction:**

Intercostal hemangioma is an extremely rare disease. It is difficult to distinguish intercostal hemangioma from other chest wall tumors. The difficulty in preoperative diagnosis may result in incomplete surgical resection and hence a high rate of recurrence.

**Case presentation:**

A 14-year-old boy who presented with asymptomatic right lateral chest wall mass with no history of trauma. Different radiological modalities were employed for diagnosis including computed tomography (CT) showed a soft tissue mass 6.5 × 4 × 5.6 cm in size abutting 5th and 6th ribs. Magnetic resonance imaging (MRI) revealed iso-intense signal in T1 and hyperintense signal in T2, that is higher than that of adjacent muscles in the inferolateral right chest wall which was compatible with intercostal hemangioma. The patient underwent surgery for excision of the mass. Through right posterolateral thoracotomy, there was a well-demarcated mass abutting 5th, and 6th ribs filling the right 5th interspaces. Histopathological examination confirmed the diagnosis of intercostal hemangioma.

**Discussion:**

Chest wall hemangiomas are uncommon and mostly arise outside the rib cage. Hemangiomas rarely occur in the intercostal space, and most of these originate from intercostal muscles (Agarwal et al., 2006). Watson and McCarthy postulated two theories for the etiology of hemangiomas. The more widely supported theory claims that hemangiomas are of congenital origin. The other theory proposes a traumatic origin of hemangiomas.

**Conclusion:**

Complete surgical resection should not be compromised by the resultant chest wall defect to prevent recurrence.

## Highlights

• Intercostal hemangioma is an extremely rare disease, accounting for approximately 0.01% of all benign hemangiomas.
• Hemangiomas are prone to bleed spontaneously or after minor traumatic injury.
• Complete excision of the tumor is mandatory even after embolization to prevent recruitment of a collatera1 blood supply.

## 1 Introduction

Intercostal hemangioma is a rare disease, accounting for approximately 0.01% of all benign hemangiomas [1]. It is difficult to distinguish intercostal hemangioma from other chest wall tumors, such as lipoma, neurogenic tumors, chondroma, fibroma and lung cancer with chest wall invasion [2]. Difficulty in preoperative diagnosis may result in incomplete surgical resection and hence a high rate of recurrence [3]. We report a child with an intercostal hemangioma presented as a chest wall tumor. Different diagnostic modalities employed for the accurate diagnosis of the tumor and successful complete surgical resection. As the patient is minor, parental consent obtained on behalf of the patient [4]. This work has been reported in line with the SCARE criteria [5].

## 2 Case report

**Fig. 1:**
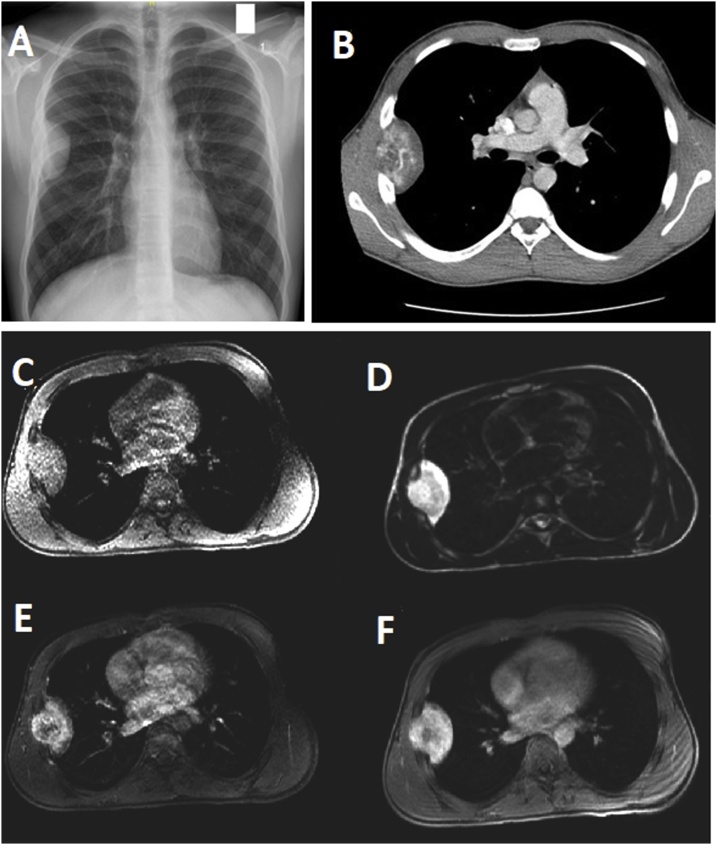
Chest X-ray with right lateral chest wall mass (A). Contrast-enhanced CT; a well-defined mass with heterogeneous enhancement arising in the 5th interspace (B). Tl-weighted image shows a solid mass with a low signal intensity but a little higher compared to that of the adjacent muscles in the right chest wall (C). The fat-suppressed T2-weighted image reveals homogeneous mass with high signal intensity similar to that of fat tissue, which was of high-intensity on fat-suppressed MRI (D). Early phases after administration of contrast, a characteristic heterogeneous enhancement of the tumor (E) that became even more uniform during the delayed phase (F).

In November 2017, an asymptomatic 14-year-old boy referred to the authors’ hospital for management of right lateral chest wall mass discovered incidently one month earlier. There was no history of trauma. Clinical examination was unremarkable. Plain chest radiography showed a smooth-walled 6.0 × 3.5 cm homogenous right lateral chest wall mass (Fig. 1A). Computed tomographic (CT) scan revealed right lateral extrapleural soft-tissue mass 6.5 × 4.0 × 5.6 cm in size abutting 5th and 6th ribs and filling the 5th intercostal space (Fig. 1B). There were neither cavitation nor calcification within the mass, and the adjacent ribs were not eroded.

Magnetic resonance imaging (MRI) revealed right extrapulmonary and extrapleural chest wall soft tissue mass. Tl-weighted MRI demonstrated a solid mass with a low signal intensity (Fig. 1C), while T2-weighted MRI demonstrated a homogeneous mass with high signal intensity similar to that of fat tissue (Fig. 1D). Early phases after administration of contrast, showed characteristic heterogeneous enhancement of the tumor that became more uniform during the delayed phase (Fig. 1E and F).

The angiogram showed a sizeable vascular mass supplied by a branch of the internal mammary artery. That branch was subsequently embolized with Gelfoam pledgets with no further filling on the post-embolization arteriogram.

**Fig. 2:**
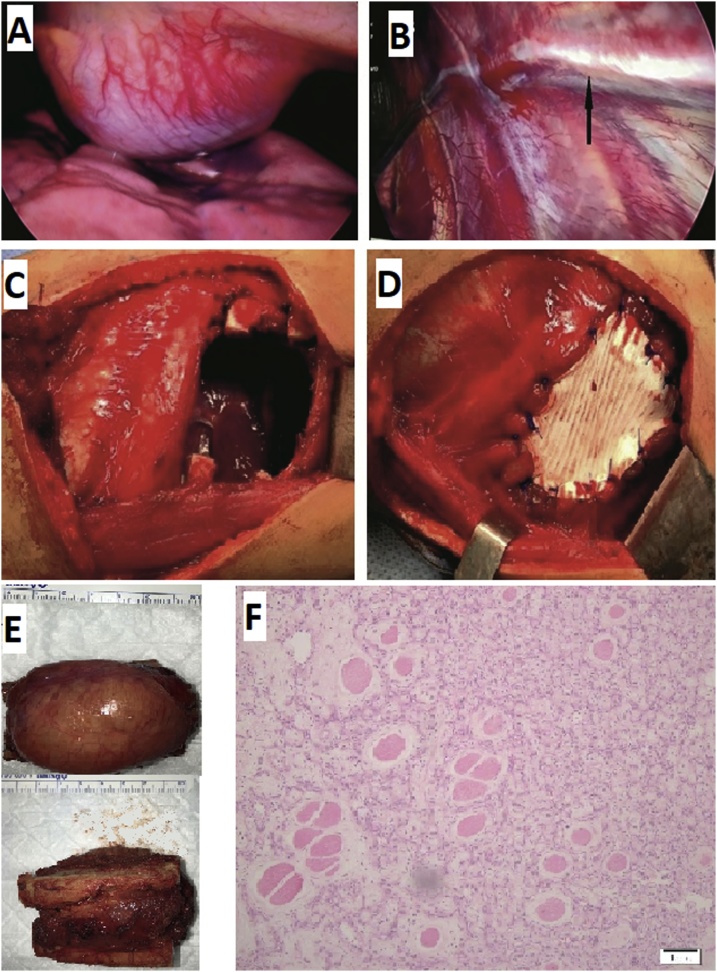
Photograph during right VAT shows large mass covered by vascular parietal pleura (A). Thrombosed feeding artery to the tumor (arrow) after embolization (B). Photograph of the chest wall defect after removal of the mass (C). Reconstruction of the chest wall defect performed using PTFE patch (D). Photograph of the specimen shows chest wall mass covered by vascular parietal pleura fixed to 5th and 6th (E). Hematoxylin and eosin stain of chest wall mass, (original magnification 10× and 20×); shows a homogeneous thick and thin-walled blood vessels with proliferating vascular spaces of capillaries of cavernous type with intervening fibrous stroma showing clusters of non-canalizing endothelial cells with no atypia nor malignancy (F).

The patient underwent an exploratory video-assisted thoracoscopy (VAT) through the right 8th intercostal space anterior axillary line (Fig. 2A and B). Posterolateral thoracotomy revealed well-demarcated mass abutting the 5th and 6th ribs filling the right 5th intercostal space. En bloc resection of the mass involved ribs and the intercostal muscles were performed (Fig. 2C). Chest wall defect was reconstructed using polytetrafluoroethylene (PTFE) patch (GORE-TEX^®^, DUALMESH^®^, W. L. Gore & Associates) (Fig. 2D).

The surgical specimen measured approximately 6 × 4 × 3.5 cm, which included a hemorrhagic, highly vascular, mass that extended from the intercostal muscles to the extra-pleural space (Fig. 2E). Cut section revealed whitish homogenous material. Microscopically, there were homogeneous thick and thin-walled blood vessels with proliferating vascular spaces of capillaries of the cavernous type with intervening fibrous stroma (Fig. 2F). There was no evidence of recurrence at 3 and six months of follow-up.

## 3 Discussion

Hemangiomas are benign slowly growing vascular tumors that account for 7% of all benign soft-tissue tumors in the whole body [4,6]. Chest wall hemangioma is part from this, and it accounts for 0.01% of all hemangiomas [1]. Chest wall hemangiomas are uncommon and mostly arise outside the rib cage. Hemangiomas rarely occur in the intercostal space, and most of these originate from intercostal muscles [7]. It is usually occurring before the age of 30, with no sex difference [8]. Watson and McCarthy postulated two theories for the etiology of hemangiomas. The more widely supported theory claims that hemangiomas are of congenital origin. The other theory proposes a traumatic origin of hemangiomas [9]. Patients with intercostal hemangioma clinically may present with palpable thoracic wall mass, the bulge in the soft-tissue, pain, or a combination of these signs and symptoms.

The preoperative diagnostic tools of chest wall tumors involve a detailed medical history, clinical examination, and radiological investigations. CT of the chest can detect the effect of the tumor on lung, ribs, and pleura, as well as phleboliths, which sometimes accompany hemangiomas [7]. Intercostal hemangioma appears as a homogeneous mass with signal intensity similar to that of skeletal muscles in Tl-weighted, and high signal intensity in T2-weighted MRI. It also shows marked enhancement by contrast in MRI in a pattern similar to that of hepatic hemangiomas on dynamic MRI. The features of dynamic imaging are the eccentric enhancement with incomplete filling peripherally in the early phase and filling during the delayed phase. These features should allow distinguishing intercostal hemangioma from the other soft tissue tumors [10].

Wide local excision with the sufficient tumor-free margin is recommended. Hemangiomas are prone to bleed spontaneously or after minor traumatic injury. Embolization of the feeding arteries ultimately followed by recurrence as the arteriovenous communications are small and numerous. Complete excision of the tumor is mandatory even after embolization to prevent recruitment of a collatera1 blood supply [11].

The expected chest wall defect should not jeopardize wide local excision of the tumor. Incomplete excision may lead to recurrence, disturbed wound healing, and secondary bleeding [12]. Reconstruction is required to restore the rigidity of the chest wall and protect the underlying structure [13]. The chest wall reconstruction is indicated whenever the resection involves more than four ribs, the defect is more than five cm and in case of anterior costal and sternal resections. The ideal prosthesis used for reconstruction should fulfill the following prerequisites; (I) rigidity to abolish paradoxical movement; (II) inertness to allow in-growth of fibrous tissue and decrease the likelihood of infection; (III) malleability to fashion to the appropriate shape at the time of operation [14]. In our patient, we used PTFE patch which provides the tension needed for a firm reconstruction that minimizes paradoxical wall movement as well as dependable suture retention. The material remains soft and highly conformable, allowing ease of handling and less irritation to surrounding tissues. The bio-prosthetic meshes is another useful material used to reconstruct the chest wall. It includes homografts, porcine intestinal submucosa, and bovine pericardium. It is used in small defects where the rigidity is not required. It is less likely to get infected and permeable to antibiotics [15].

Methyl Methacrylate has been the best choice to reconstruct the sternum, ribs and chest wall, entirely or partially for many years. It is usually sandwiched between two layers of the mesh to strengthen the rigidity of the reconstruction. It is suitable for most of extensive anterior and lateral chest wall defects, prevents paradoxical movements and chest deformities. However, methylmethacrylate material is not permeable to fluids and therefore, are considered to increase pain and excessive chest wall rigidity, liable to fracture and has a higher risk of infection [16].

Titanium plates are newly introduced material that seems to be an ideal prosthetic solution for chest wall reconstruction. It has resistance to corrosion, a low weight, resistance to traction, compatible with MRI, and biologically inert [15]. Many authors agree that the titanium system represents a better solution in the reconstruction of large full-thickness defects to support the chest wall after demolition for neoplastic disease [17,18]. It restores the rigidity of the thoracic cage and prevents respiratory and infective complications in neoplastic cases. The reconstruction of the chest wall requires integration with traditional techniques, such as synthetic biologic or titanium meshes and various muscle flaps [15]. Bar fracture and plate dislocation are the two most frequent complications of the titanium plates. Bar fracture rate varied from 0 to 11% in some series, and plate dislocation frequently is due to the mismatch between the screws length and rib thickness, or the destruction of the bone threads that lock the screws into the rib [19].

## 4 Conclusion

This paper presented a case of intercostal hemangioma arising from the right lateral 5th intercostal muscles. A tentative diagnosis is feasible due to the characteristic imaging of the tumor. Complete surgical resection should not be compromised by the resultant chest wall defect to prevent recurrence.

## Conflicts of interest

No conflict of interest to declare.

## Sources of funding

No source of funding.

## Ethical approval

Institutional Review Board approval. Case Report is presented anonymously.

## Consent

Patient is a minor, we have parental consent on behalf of the patient.

## Author’s contribution

Hatem Elbawab: Study concept, writing the paper.

Farouk Alreshaid: Writing the paper.

Asayil Alnasser: Editing the paper.

Raja Husain: Communication, submission.

Yasser Aljehani: Writing the paper.

Tarek Hashem: pathologist, pathology report writing.

## Registration of research studies

Not Applicable (Case Report; not an interventional study).

## Guarantor

Hatem Elbawab.

## Provenance and peer review

Not commissioned, externally peer-reviewed.
